# Using a multi‐model ensemble approach to determine biodiversity hotspots with limited occurrence data in understudied areas: An example using freshwater mussels in México

**DOI:** 10.1002/ece3.8909

**Published:** 2022-05-13

**Authors:** Alexander H. Kiser, Kevin S. Cummings, Jeremy S. Tiemann, Chase H. Smith, Nathan A. Johnson, Roel R. Lopez, Charles R. Randklev

**Affiliations:** ^1^ Texas A&M AgriLife Research Center at Dallas Texas A&M Natural Resources Institute Dallas Texas USA; ^2^ Illinois Natural History Survey Urbana‐Champaign Illinois USA; ^3^ Department of Integrative Biology University of Texas Austin Texas USA; ^4^ U.S. Geological Survey, Wetland and Aquatic Research Center Gainesville Florida USA; ^5^ Natural Resources Institute Texas A&M University College Station Texas USA

**Keywords:** climate, conservation, habitat, maxent, mycetopodidae, random forest, species distribution model, unionidae

## Abstract

Species distribution models (SDMs) are an increasingly important tool for conservation particularly for difficult‐to‐study locations and with understudied fauna. Our aims were to (1) use SDMs and ensemble SDMs to predict the distribution of freshwater mussels in the Pánuco River Basin in Central México; (2) determine habitat factors shaping freshwater mussel occurrence; and (3) use predicted occupancy across a range of taxa to identify freshwater mussel biodiversity hotspots to guide conservation and management. In the Pánuco River Basin, we modeled the distributions of 11 freshwater mussel species using an ensemble approach, wherein multiple SDM methodologies were combined to create a single ensemble map of predicted occupancy. A total of 621 species‐specific observations at 87 sites were used to create species‐specific ensembles. These predictive species ensembles were then combined to create local diversity hotspot maps. Precipitation during the warmest quarter, elevation, and mean temperature were consistently the most important discriminatory environmental variables among species, whereas land use had limited influence across all taxa. To the best of our knowledge, our study is the first freshwater mussel‐focused research to use an ensemble approach to determine species distribution and predict biodiversity hotspots. Our study can be used to guide not only current conservation efforts but also prioritize areas for future conservation and study.

## Graphical abstract

This research article evaluates the utility of multi‐model ensemble species distribution models (ESDMs) to predict occupancy in regions where there is limited distribution and environmental data. Our study focuses on freshwater mussels, which are globally imperiled, in the Pánuco River Basin in central México, a region considered one of 25 biodiversity hotspots in the world. We identified climate and landscape factors that were helpful for explaining occupancy and should be useful for conservationists in this region interested in better understanding how climate change and flow contribute to mussel persistence.

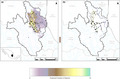

## 1 INTRODUCTION

Understanding processes and constraints influencing the distribution and abundance of species is a fundamental goal of basic and applied ecological research (Austin, 2002; Jones & Lawton, 2012; Poff, 1997). Of particular interest over the last few decades has been understanding how the spatial and temporal configuration of habitat shapes ecological processes (Newton et al., 2008). In lotic systems, habitat is viewed as a hierarchy with stream segments, reaches, mesohabitats, and microhabitats nested within a watershed. These habitat levels are simultaneously parts and wholes (Miller, 2008), such that each level shapes the environment of all habitat levels nested within it (Frissell et al., 1986).

Poff (1997) recognizing the hierarchical nature of environmental constraints and the role it plays in shaping the distribution and abundance of lotic species developed a conceptual framework contrasting species traits with multi‐scale habitat data after. The resulting model acknowledged that each habitat level has its own functional and structural properties shaped by the previous level, which served as environmental filters of species traits over varying spatiotemporal periods. In practice, this idea suggests a species can only be present at one level of habitat (i.e., mesohabitat scale) if it is able to pass through preceding habitat levels, each having its own unique properties that filter taxa lacking certain prerequisite traits (Poff, 1997; Southwood, 1977, 1988). Inoue et al. (2017) demonstrated this point by evaluating the distribution of freshwater mussels and fish in central and northern Europe, where species occupancy was determined, in part, by a suite of nested environmental variables ranging from landscape to the mesohabitat scale. Similar findings have been observed for other taxa, such as reef fish assemblages (López‐Pérez et al., 2013), forest plant communities (Kolb & Diekmann, 2004), and wetland species (Quesnelle et al., 2013). These studies suggested a hierarchical perspective of habitat configuration might be useful to inform basic and applied ecological research.

Species distribution models (SDMs) have become an increasingly useful tool for understanding how habitat filters (i.e., land use, topography, and climate) shape species distributions. Because of this utility, SDMs have been widely applied to estimate species ranges, identify environmental factors shaping distribution, abundance patterns, determine areas of conservation importance, and predict ranges under past and future environmental scenarios (Daniel et al., 2018; Dormann, 2007; Thuiller, 2003; Wilson et al., 2011). SDMs are rooted in the concepts presented by Poff (1997), as well as niche theory, which proposes the distribution of a species is related to its ability to cope with varying environmental conditions (Grinnell, 1917; Hutchinson, 1957; Peterson et al., 1999). SDMs work by relating environmental and biological data to species presence to determine habitat limits, which are subsequently used to predict occupancy or environmental suitability across a geographic area (Buckley et al., 2010; Guisan & Thuiller, 2005). Because SDMs are based on multi‐scaled habitat data, predicted ranges for a given species represent geographic areas across time and space that permit species occurrence.

Ensemble SDMs (ESDMs), a type of SDM, have become an increasingly popular approach to predicting the occupancy of rare species (De Marco & Nóbrega, 2018; Sousa‐Silva et al., 2014). Ensemble modeling works by taking individual SDMs (e.g., MAXENT, Random Forest, Boosted Regression Trees, etc.) and averaging their output into one final prediction, which greatly reduces the generalization error of single model approaches (Araújo & New, 2007; Hao et al., 2019; Seni & Elder, 2010). Individual models can be combined in a variety of ways, with the simplest being the mathematical mean or median (Hao et al., 2019), while more complex options include weighted averages, or scaling predictions based on model evaluation statistics (Araújo & New, 2007; Hao et al., 2019; Marmion et al., 2009). The combined predictions, regardless of the approach, are often better than standalone SMD methods. Hao et al. (2019) found through a meta‐analysis of SDM studies that ensemble model performance was generally higher than individual models. Elder and Lee (1997) comparing the model potential of ensembles versus single model type found predicted occupancy was similar between ensemble and single models, but ensembles had lower out‐of‐the‐bag error (mean prediction error of training data). Seni and Elder (2010), building off Elder and Lee (1997), noted ensemble models were generally better than single models because of weighted averages, model selection, and variable pruning to optimize total model performance. Additionally, ESDMs distribute individual model type bias more evenly and reduce overprediction bias based on data types (i.e., continuous, binomial, and categorical), which creates less disjointed and abrupt predicted ranges compared to single‐model methods.

In this study, we use ESDMs to predict distributions of an imperiled and understudied group of freshwater bivalves native to central and southern México. This region harbors some of the most biologically diverse freshwater streams in the world (Graf & Cummings, 2021a; Smith & Bermingham, 2005). The Pánuco River Basin (hereafter PRB), located in East Central México, is one of these diverse drainages, and contains more than 95 species of fish, dozens of species of freshwater mollusks (gastropods and bivalves), and a diverse collection of other macroinvertebrates (Martínez‐Lendech et al., 2020). Included among the mollusks are 14 species of native freshwater mussels, eight of which are endemic to the Pánuco River Basin and one introduced species. The status and distribution of freshwater mussels in the PRB, including factors that contribute to their persistence, remain unknown. As a result, it is unclear how habitat at various spatial and temporal scales shapes the distribution and abundance of the mussel fauna in the PRB or how species traits facilitate occupancy at those various scales. It is also unclear if species distributions and composition have changed overtime. The lack of accurate distribution and composition data can be problematic and will likely negatively affect the conservation and management of the mussel fauna in this region.

To begin addressing the knowledge gaps for mussels within the PRB, we evaluated the role of habitat in shaping mussel occurrence across the landscape. We also provide baseline information for a fauna that is poorly known in a region considered as one of the 25 major global biodiversity hotspots in need of conservation, protection, and further study (Myers et al., 2000). Additionally, we assess the potential of ESDMs for predicting occurrence with limited environmental and presence data for conservation and management of species. The specific objectives of our study were to (1) use SDMs and ESDMs to predict the distribution of freshwater mussels in the PRB; (2) determine habitat factors shaping mussel occurrence in the PRB; and (3) use predicted occupancy across a range of taxa to identify mussel biodiversity hotspots to guide conservation and management.

## 2 METHODS

### 2.1 Study area, focal taxa, and environmental data

The PRB is the 3rd largest river basin by size (98,227 km^2^) in México and contains three major sub‐basins, the Moctezuma (42,726 km^2^), Tamuin (also called the Tampaón) (33,260 km^2^), and Tamesí (19,127 km^2^, Hudson et al., 2005). The Pánuco River is the primary stream within the PRB and is considered the 10th longest river in México (510 km) and the 4th largest by discharge (20.3 billion m^3^/annually, Hudson, 2003. The Pánuco River is formed from the Moctezuma and Tamuin rivers after crossing the Sierra Madre Oriental and joining with the Tamesí River north of Tampico, México, before discharging into the Gulf of México (Hudson et al., 2005). The PRB is located primarily in a subtropical climate zone with annual precipitation of approximately 30 cm near the headwaters, increasing to 240 cm near the coast (Hudson, 2003). Rainfall is typically greatest from May to October, with July and September being the wettest months. Temperature varies greatly with elevation, but on average ranges from 15°C in January to 24°C in June (Hudson, 2003). Land use within the PRB is largely farming and ranching with primary crops being sugarcane, citrus, and coffee (Hudson et al., 2005).

**FIGURE 1:**
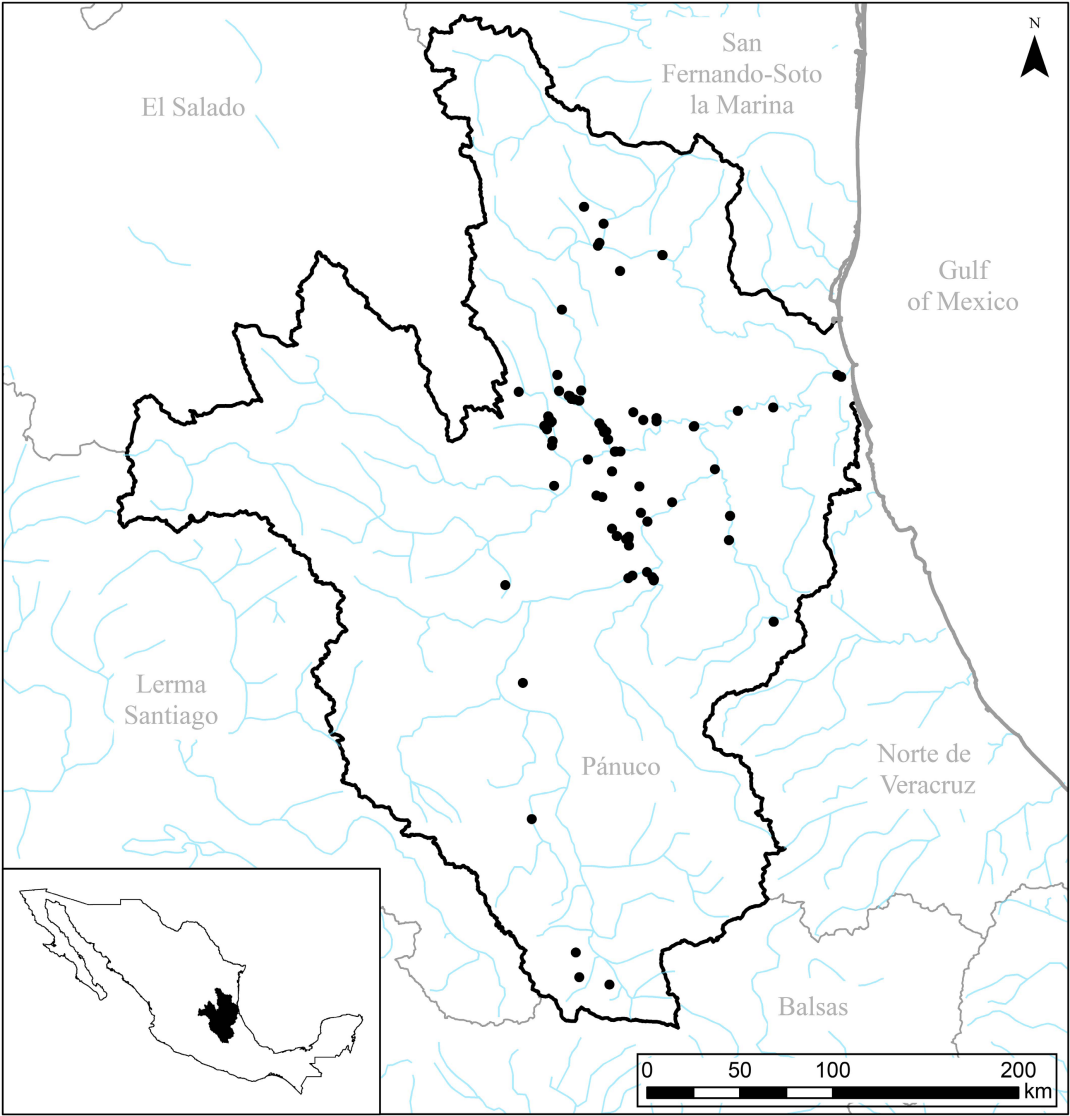
Map of the Pánuco River Basin. Black circles denote presence records for the following focal species: *Actinonaias coyensis*, *Actinonaias medellina*, *Actinonaias signata*, *Disconaias disca*, *Disconaias fimbriata*, *Cyrtonaias tampicoensis*, *Friersonia iridella*, *Nephronaias aztecorum*, *Popenaias berezai*, *Popenaias semirasa*, and *Anodontites cylindracea*. Presence data includes recent and historical observations collected by Texas A&M, Illinois Natural History Survey, Urbana‐Champaign, MUSSELp, and U.S. Geological Survey. Denoted presence may represent multiple species within the same locale

Occurrence data includes recent and historical observations collected by Texas A&M University, the Illinois Natural History Survey, Urbana‐Champaign, MUSSELp (http://mussel‐project.uwsp.edu/), and U.S. Geological Survey. Nomenclature for mussels followed Graf and Cummings (2021a). We focused on 15 species found in the PRB (Figure 1) whose species relationships and distribution remain unresolved (Graf & Cummings, 2021a). Occurrence data for our focal species came from surveys conducted in 2017 and 2018 (Inoue et al., 2020) and from data aggregated by MUSSELp (Graf & Cummings, 2021b). In total, we obtained 621 records across 87 sites within the PRB of the following species: *Utterbackia imbecillis*, *Actinonaias coyensis*, *Actinonaias medellina*, *Actinonaias signata*, *Disconaias disca*, *Disconaias fimbriata*, *Cyrtonaias tampicoensis*, *Friersonia iridella*, *Nephronaias aztecorum*, *Popenaias berezai*, *Popenaias semirasa*, *Psoronaias semigranosa*, *Megalonaias nickliniana*, *Anodontites cylindracea*, and *Anodonta impura* (Table 1). Duplicate observations of the same species at the same location and those species with <10 unique observations were omitted due to poor model success.

**TABLE 1:** Total number of observations by species and the number of non‐duplicate locations used in the training and testing of models

Order	Super family	Family	Subfamily	Tribe	Genera	Species	Total observations	Unique spatial observations	Pánuco Basin Endemic
Unionoida									
	Unionoidea								
		Unionidae							
			Unioninae						
				Anodontini					
					*Anodonta*				
						*Anodonta impura* (Say, 1829)	5	5	
					*Utterbackia*				
						*Utterbackia imbecillis* (Say, 1829)	2	2	
			Ambleminae						
				Lampsilini	*Actinonaias*				
						*Actinonaias coyensis* (Pilsbry, 1910)	43	23	x
						*Actinonaias medellina* (Lea, 1838)	17	10	
						*Actinonaias signata* (Pilsbry, 1910)	79	24	x
					*Disconaias*				
						*Disconaias disca* (Lea, 1838)	33	15	x
						*Disconaias fimbriata* (Frierson, 1907)	67	28	
					*Crytonaias*				
						*Cyrtonaias tampicoensis* (Lea, 1838)	34	20	
					*Friersonia*				
						*Friersonia iridella* (Pilsbry & Frierson, 1908)	134	43	x
				Popenaiadini	*Nephronaias*				
						*Nephronaias aztecorum* (Philippi, 1847)	21	13	
					*Popenaias*				
						*Popenaias berezai* (Inoue et al., 2020)	99	39	
						*Popenaias semirasa* (Pilsbry, 1910)	60	10	x
					*Psoronaias*				
						*Psoronaias semigranosa* (von dem Busch in Philippi, 1843)	11	5	
				Quadrulini	*Megalonaias*				
						*Megalonaias nickliniana* (Lea, 1834)	5	3	
	Etherioidea								
		Mycetopodidae							
			Anodontitinae						
					*Anodontites*				
						*Anodontites cylindracea* (Lea, 1838)	11	10	

To model the distribution of our focal species, we used climate, elevation, and land use data. We chose these variables because previous studies have shown all three are important determinants of mussel occupancy (Gama et al., 2016; Hopkins, 2009; Santos et al., 2015) and because these data were readily available within our remote study area. Climate data were obtained from the Worldclim bioclimatic database (Fick & Hijmans, 2017) and include data on the minimum and maximum levels, mean values and ranges, and quarterly summaries for temperature and precipitation on a global scale (Hijmans et al., 2005; Maria & Udo, 2017; Nix, 1986). We also used air temperature as a proxy for water temperature (Caissie, 2006; Mohseni & Stefan, 1999). Elevation data were taken from WorldClim, and land cover data were obtained from DIVA‐GIS (Hijmans et al., 2004), both of which were taken at the same resolution and extent as the Worldclim bioclimatic data. Percent slope changes were calculated from the elevation data set using the terrain function in the “raster” package of R (Hijmans, 2020). Multicollinearity was evaluated using Pearson correlation and covariates with values greater than 0.8 were excluded from further analysis (Dormann et al., 2013). The resulting covariates were selected with a grid cell resolution size of 30 arc seconds (roughly 0.9 km): BIO2 = Mean Diurnal Range, BIO3 = Isothermality, BIO8 = Mean Temperature of Wettest Quarter, BIO9 = Mean Temperature of Driest Quarter, BIO15 = Precipitation seasonality, BIO18 = Precipitation of Warmest Quarter, BIO19 = Precipitation of Coldest Quarter, Alt = Elevation, Layer = Landcover and use, and Percent Slope Change.

### 2.2 Modeling protocol

To predict mussel occurrence in the PRB, we used an ensemble modeling approach. In our analysis, we used the following models, which are all available within the “SDM” package in R (Naimi & Araújo, 2016): General Linear Model (GLM), Support‐Vector Model (SVM), Random Forest (RF), Boosted Regression Tree (BRT), Multivariate Adaptive Regression Splines (MARS), Maximum Entropy (MAXENT), Classification and Regression Trees (CART), Flexile Discriminate Analysis (FDA), and Mixture Discriminant Analysis (MDA). Other models available within the “SDM” package were evaluated and removed due to insufficient data or poor overall performance compared to other model types (AUC < 0.8, TSS < 0.7, see below). The combined dataset with historical and contemporary records was used to populate presence records for our focal species. Absence data were unavailable due to the exploratory nature of the surveys conducted within the PRB. Because of this, following Liu et al. (2013), we randomly generated pseudo‐absences at two times the number of known presence observations of each species within the extent of the PRB.

To train the individual models, we used 80% of the occurrence records for a given species to develop predictions, which were then tested against the remaining 20% of data. To improve parameter estimates, each training and testing group was randomly resampled using 100 bootstrap replicates. Both individual model methods that were used to create the final ensemble model and the ensemble model for each species were assessed using the true skill statistic (TSS) and area under the curve (AUC) of the receiver operating characteristic (ROC). TSS takes into consideration both omission (false absences) and commission (false presences) errors and is unaffected by prevalence (Allouche et al., 2006). TSS ranges from 0 to 1, and values from 0.2 to 0.5 indicate poor model fit, those values from 0.6 to 0.8 denote adequate model fit, and values greater than 0.8 are considered excellent model fit (Coetzee et al., 2009). The ROC shows the classifying performance based on a threshold parameter (Fielding & Bell, 1997; Phillips et al., 2004). It plots the true positive rate against the false‐positive rate relative to each threshold, creating a curve of expected outcomes. The area under the ROC, or AUC is the probability of a model classification correctly predicting the outcome (i.e., presence vs. absence). Models with AUC values <0.5 are considered worse than random, values from 0.5 to 0.7 are considered poor, 0.7–0.9 are considered fair, and values >0.9 are considered excellent fit (Swets, 1988). In addition to model performance, the variable of importance, which identifies the variable that contributes the most to model accuracy, was also assessed using AUC and Pearson Rank Correlation (PRC). PRC measures the correlation between the predicted value and error of a model when a random variable is permutated, and all other variables are held at their mean. The decline in model accuracy from a variable permutation while all other variables are held at their mean can then be used to determine the variable importance to overall model accuracy and to determine key factors affecting species presence and absence (Thuiller et al., 2009).

To map the predicted presence of a given species within a cell (30 arc‐second grid), optimum threshold (OT) values for the species were created from the weighted average based on the maximum (sensitivity + specificity) of all species‐specific models. Predicted values of a cell greater than the OT of a species were given a value of 1 to indicate predicted presence, and 0 for predicted absence. These binomial presence/absence ensemble maps for each species were then stacked by summing the collected outputs to create local richness maps which denote the number of predicted species within a given cell. All statistical analysis was performed using R version 4.1.1 (R Core Team, 2021).

## 3 RESULTS

A total of 11 species with 621 species‐specific observations at 87 unique sites were used in our ensemble modeling after removing species with small sample sizes (i.e., *n* ≤ 10: *U*. *imbecillis* (2), *P*. *semigranosa* (5), *M*. *nickliniana* (3), and *A*. *impura* (5)) and duplicate occurrences. *F*. *iridella* (43), had the greatest number of occurrences followed by *P*. *berezai* (39), *D*. *fimbriata* (28), *A*. *signata* (24), *A*. *coyensis* (23), *C*. *tampicoensis* (20), *D*. *disca* (15), *N*. *aztecorum* (13), *A*. *medellina* (10), *Popenaias s*. (10), and *A*. *cylindracea* (10). Model completion was 100% for all model types except the BRT model which would not consistently run for species with less than 20 observations consistently.

**FIGURE 2:**
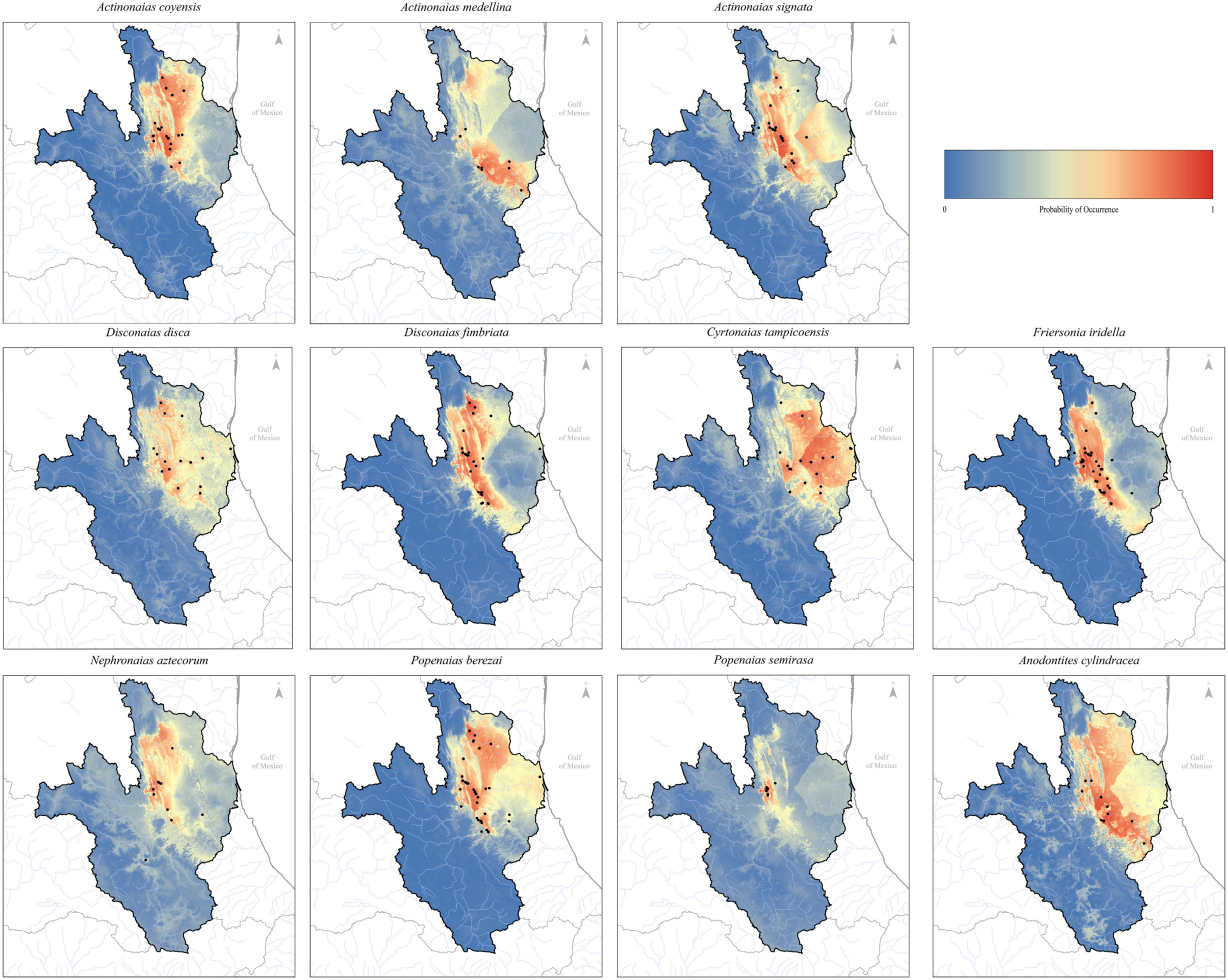
Species‐specific ensemble model outputs of 11 focal species within the Pánuco River Basin. Weighted averages of nine individual species distribution model methodologies (General Linear Model, Support‐Vector Model, Random Forest, Boosted Regression Tree, Multivariate Adaptive Regression Splines, Maximum Entropy, Classification and Regression Trees, Flexile Discriminate Analysis, and Mixture Discriminant Analysis) based on maximum (sensitivity + specificity). Red indicates a higher probability of occurrence for a given cell (30 arc‐second grid, ~0.9 km)

For all individual type model outputs (Figure 2), model performance was fair to excellent based on testing parameters (Table 2). For TSS, model accuracy for 8 out of 11 species indicated excellent fit (TSS > 0.8) across all models. For AUC, 8 of 11 species specific model methods were greater than 0.9, indicating excellent fit. *D*. *disca* had the lowest overall model performance (mean 0.829 AUC ± 0.06, 0.700 TSS ± 0.08) but is still in the good fit range for model accuracy (AUC > 0.8, TSS > 0.7). The model type that performed best overall across all species was RF (0.962 AUC ± 0.03, TSS 0.908 ± 0.05), followed by MAXENT (0.936 AUC ± 0.03, TSS 0.867 ± 0.06), SVM (0.933 AUC ± 0.05, TSS 0.86 ± 0.07), BRT (0.928 AUC ± 0.02, TSS 0.813 ± 0.02), MDA (0.905 AUC ± 0.03, TSS 0.805 ± 0.04), FDA (0.9 AUC ± 0.05, TSS 0.81 ± 0.07), MARS (0.885 AUC ± 0.05, TSS 0.823 ± 0.08), CART (0.877 AUC ± 0.04, TSS 0.731 ± 0.07), and lastly the lowest performing model was the GLM model GLM (0.872 AUC ± 0.04, TSS 0.79 ± 0.07). Based on our assessment statistics, all models performed in the good to excellent range.

**TABLE 2:** Individual species distribution model performance for each species of freshwater mussels found within the Pánuco River Basin, México

	*Actinonaias coyensis*			*Actinonaias medellina*			*Actinonaias signata*					
Model	AUC	TSS	Deviance	AUC	TSS	Deviance	AUC	TSS	Deviance			
GLM	0.89	0.82	7.05	0.88	0.85	7.43	0.87	0.76	7.17			
SVM	0.95	0.89	0.67	0.96	0.93	0.69	0.94	0.84	0.66			
RF	0.98	0.94	0.41	0.97	0.94	0.53	0.97	0.9	0.46			
BRT	0.91	0.79	0.87	NA	NA	NA	0.93	0.82	0.84			
MARS	0.93	0.88	3.74	0.91	0.88	5.67	0.89	0.83	5.89			
MAXENT	0.96	0.91	0.54	0.95	0.93	0.66	0.95	0.86	0.64			
CART	0.91	0.77	1.01	0.87	0.74	1.06	0.9	0.75	1.03			
FDA	0.9	0.8	1.13	0.92	0.87	3.32	0.91	0.81	0.99			
MDA	0.92	0.84	1.74	0.89	0.79	2.05	0.93	0.84	1.51			

	*Disconaias disca*			*Disconaias fimbriata*			*Cyrtonaias tampicoensis*			*Friersonia iridella*		
Model	AUC	TSS	Deviance	AUC	TSS	Deviance	AUC	TSS	Deviance	AUC	TSS	Deviance
GLM	0.78	0.64	12.42	0.89	0.81	6.76	0.83	0.71	10.06	0.93	0.84	2.75
SVM	0.83	0.71	1.18	0.95	0.88	0.59	0.89	0.81	0.93	0.95	0.84	0.64
RF	0.92	0.83	0.73	0.98	0.94	0.36	0.93	0.87	0.62	0.98	0.9	0.4
BRT	NA	NA	NA	0.95	0.84	0.79	0.91	0.8	0.88	0.95	0.83	0.74
MARS	0.77	0.64	12.85	0.94	0.9	3.56	0.84	0.75	9.16	0.91	0.83	5.38
MAXENT	0.89	0.77	0.94	0.97	0.9	0.54	0.89	0.81	0.7	0.96	0.86	0.55
CART	0.79	0.58	1.76	0.91	0.78	0.96	0.86	0.7	1.47	0.91	0.77	0.9
FDA	0.79	0.66	2.42	0.94	0.84	1.05	0.88	0.77	1.25	0.94	0.84	0.77
MDA	0.86	0.75	5.87	0.96	0.88	1.44	0.89	0.8	2.46	0.95	0.85	0.95

	*Nephronaias aztecorum*			*Popenaias berezai*			*Popenaias semirasa*			*Anodontites cylindracea*		
Model	AUC	TSS	Deviance	AUC	TSS	Deviance	AUC	TSS	Deviance	AUC	TSS	Deviance
GLM	0.82	0.74	10.07	0.9	0.79	3.67	0.89	0.85	7.03	0.91	0.88	5.85
SVM	0.89	0.8	0.96	0.94	0.84	0.64	0.98	0.96	0.5	0.98	0.96	0.54
RF	0.91	0.86	0.77	0.97	0.89	0.45	1	0.99	0.31	0.97	0.93	0.53
BRT	NA	NA	NA	0.92	0.8	0.83	NA	NA	NA	NA	NA	NA
MARS	0.84	0.77	8.58	0.89	0.81	6.64	0.94	0.92	4.57	0.88	0.84	7.13
MAXENT	0.89	0.8	0.91	0.94	0.85	0.58	0.96	0.94	0.75	0.94	0.91	0.71
CART	0.82	0.62	1.47	0.9	0.77	1.04	0.92	0.84	0.72	0.86	0.72	1.37
FDA	0.85	0.77	3.45	0.9	0.78	0.92	0.93	0.89	4.15	0.94	0.88	4.93
MDA	0.86	0.76	8.74	0.91	0.8	1.29	0.9	0.8	3.57	0.88	0.75	4.08

Note

Each individual species distribution methodology (General Linear Model (GLM), Support‐Vector Model (SVM), Random Forest (RF), Boosted Regression Tree (BRT), Multivariate Adaptive Regression Splines (MARS), Maximum Entropy (MAXENT), Classification and Regression Trees (CART), Flexile Discriminate Analysis (FDA), and Mixture Discriminant Analysis (MDA)) were bootstrapped 100 times. Performance metrics are based on area under the curve (AUC), true skill statistics (TSS), and model deviance.

All species ensemble models had an AUC > 0.85 and TSS > 0.86, indicating good to excellent predictive fit (Table 3). Sensitivity (correctly predicted presence) was >0.84 and specificity (correctly predicted absences) was >0.84 for all species, indicating accurate model prediction power. The variables of importance were similar across species with elevation, precipitation in the warmest quarter, and mean temperature during the driest quarter were the most influential variables (Table 4), although the order and degree of importance varied among species. For most species, the land cover was uninformative (Table 4), that is, predictive power was unaffected by its removal, which is likely due to the coarse‐scale (30 arc‐seconds), or the homogeneity of landscape (agriculture) within the region.

**TABLE 3:** Summary of the following ensemble model performance parameters by species: AUC, TSS, sensitivity and specificity, and weighted average optimal threshold values based on the maximum (sensitivity + specificity)

Species	AUC	TSS	Sensitivity	Specificity
*Actinonaias coyensis*	0.935	0.939	0.932	0.933
*Actinonaias medellina*	0.932	0.938	0.924	0.932
*Actinonaias signata*	0.928	0.932	0.927	0.926
*Disconaias disca*	0.851	0.867	0.843	0.844
*Disconaias fimbriata*	0.947	0.950	0.947	0.946
*Cyrtonaias tampicoensis*	0.888	0.895	0.886	0.886
*Friersonia iridella*	0.944	0.946	0.944	0.944
*Nephronaias aztecorum*	0.886	0.900	0.879	0.876
*Popenaias berezai*	0.923	0.926	0.922	0.923
*Popenaias semirasa*	0.953	0.960	0.946	0.953
*Anodontites cylindracea*	0.934	0.944	0.929	0.935

**TABLE 4:** Variable of importance by species for a given ensemble model

	*Actinonaias coyensis*		*Actinonaias medellina*		*Actinonaias signata*			
Variables	COR	AUC	COR	AUC	COR	AUC		
Diurnal range	19.4	14.4	20.2	16.1	20.7	13.9		
Temp seasonality	21.9	17.4	20	16.2	27.1	22.8		
Mean temp of wettest quarter	25.2	19.6	31.7	26.5	22.3	13.1		
Mean temp of driest quarter	17.6	12.3	19.3	14.5	35.1	30.3		
Precip seasonality	7.1	4.9	13.2	8.9	13.9	9.2		
Precip of warmest quarter	27.6	20.9	26.1	23	30.3	24.4		
Precip of coldest quarter	13.6	8.4	16.8	11.8	17.2	11.3		
Elevation	32.1	27.2	32.5	29.6	33.8	28.9		
Landcover	6.3	4.1	9.2	5.6	4.5	2.3		
Percent slope change	7.1	5.4	8.7	6.2	4.2	2.3		

	*Disconaias disca*		*Disconaias fimbriata*		*Cyrtonaias tampicoensis*		*Friersonia iridella*	
	COR	AUC	COR	AUC	COR	AUC	COR	AUC
Diurnal range	24.4	15.4	15.6	9.4	19.1	11.8	16.1	10.2
Temp seasonality	21	13.4	22.5	19.3	23	16.7	25.7	21.6
Mean temp of wettest quarter	21.5	12.4	17	12	20.3	12.5	16.3	11.4
Mean temp of driest quarter	21.6	12.7	12.1	8.1	37.3	30.2	10	6.5
Precip seasonality	14.1	7.3	5.4	3.5	15	8.5	4.2	2.5
Precip of warmest quarter	30.2	21.7	45	36.9	19.8	12.8	46.9	37
Precip of coldest quarter	19.3	10	10.7	6.4	18.2	10.3	12.2	7.9
Elevation	31.9	25	33.9	30.5	34.9	27.5	33.4	30.5
Landcover	15.5	11	4.3	2.5	9.9	6.4	3	1.8
Percent slope change	15.2	8.8	3.7	1.9	8.5	5.2	4.1	2.4

	*Nephronaias aztecorum*		*Popenaias berezai*		*Popenaias semirasa*		*Anodontites cylindracea*	
	COR	AUC	COR	AUC	COR	AUC	COR	AUC
Diurnal range	21.9	15.3	13.7	8.2	12.9	9.2	16.1	12.9
Temp seasonality	21.6	15.6	19.8	14	17.6	14.9	14	11.2
Mean temp of wettest quarter	25.3	18.9	16.7	11.6	18.8	14.9	25.1	22.1
Mean temp of driest quarter	25.9	20	14.7	10.2	24	21.5	16	12.7
Precip seasonality	16.9	10.6	9.4	6	11.7	7.9	6.6	4.4
Precip of warmest quarter	46.4	39.1	28.5	21.2	46.8	42.1	19.8	16.7
Precip of coldest quarter	23.1	14.4	12.1	6.8	14.2	10.3	12	8.8
Elevation	29.5	23.7	37.9	32.5	20.9	17.7	35.1	33
Landcover	11.1	7.2	4.9	2.3	8.1	4	14.4	9.3
Percent slope change	8.9	5.1	4.2	2.2	6.1	4.5	8.9	6.7

Note

Pearson Rank Correlation (Pearson) and area under the curve (AUC) were used to evaluate variable importance with higher numbers for each statistic indicating an increasing level of significance to model accuracy. All numbers are percentages.

**FIGURE 3:**
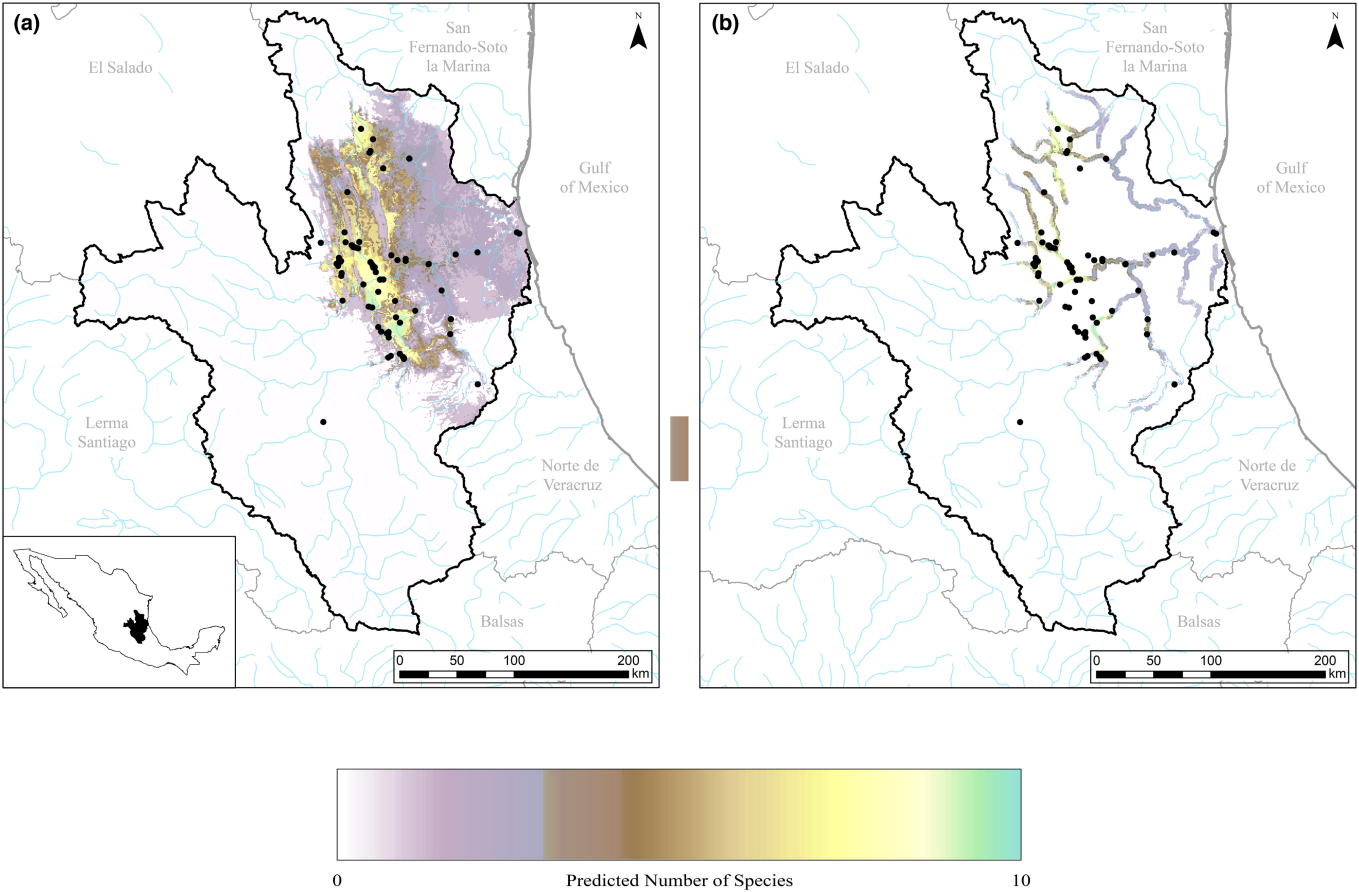
(a) Map of local biodiversity hotspots within the entire Pánuco River Basin; (b) biodiversity map outputs restricted to within 2 km buffer of major streams within the basin. Biodiversity maps were created using stacked ensemble model distributions for 11 species of freshwater mussels found within the reach: *Actinonaias coyensis*, *Actinonaias medellina*, *Actinonaias signata*, *Disconaias disca*, *Disconaias fimbriata*, *Cyrtonaias tampicoensis*, *Friersonia iridella*, *Nephronaias aztecorum*, *Popenaias berezai*, *Popenaias semirasa*, and *Anodontites cylindracea*. Black circles denote known presence data from all species modeled and may represent multiple species within the same location

The stacked predictive map illustrated a high likelihood of multiple species within the PRB (Figure 3). Locations with the greatest number of species (*n* = 10) were in the central and eastern portions of the basin along the steppe region transitioning from the Central México Plateau to the coastal plain. Specifically, west of the Ciudad Valles region and north of Ciudad Mante all had a high predicted diversity of more than eight species. Many of these hotspots coincided with locations where mussels were known to occur, which indicates an accurate model fit (AUC > 0.9, TSS > 0.8). However, there were also instances where predicted diversity was shown to be high (>8) in locations that had not been sampled. The tributaries to the Pánuco River, around the villages of Loma Alta and La Isla de Ocampo in the northern portion of the basin in Tamaulipas are one of these locations and showed a high probability of harboring multiple species (*n* = 8) with limited sampling in the area, making this region a high priority for future sampling.

## 4 DISCUSSION

In this study, we modeled distributions of some of the mussel species found in the PRB, understudied drainage in East Central México, using a limited suite of available environmental and climatic variables. We accomplished this using an ensemble approach, wherein multiple SDM methodologies were combined to create a single ensemble map of predicted occupancy. To the best of our knowledge, this is the first study to use an ensemble approach to determine the species distribution of freshwater mussels and predict biodiversity hotspots. Of the variables assessed in our study, precipitation during the warmest quarter, elevation, and mean temperature during the driest quarter were consistently the most important discriminatory environmental variables among species, whereas land use had limited influence across all taxa. We suggest these findings can be used to guide current conservation efforts and prioritize objectives for future conservation planning for freshwater mussels in the PRB.

### 4.1 Ensemble species distribution models

SDMs are an important component of conservation and natural resource management for the game and non‐game species (Elith & Leathwick, 2009; Randklev et al., 2015; Sherwood et al., 2018). In the United States, the U.S. Fish & Wildlife Service (USFWS) has used ensemble models to support species conservations status assessments because of its predictive power, accuracy, and ability to handle understudied species and/or areas with limited environmental data (Breiner et al., 2015; Burnham & Anderson, 2002; Marmion et al., 2009). Hao et al. (2020) found ensemble models outperformed untuned individual models (i.e., those models unadjusted to obtain optimal performance), and when predicting distant areas, tuned individual SDMs had similar performance to ensembles. However, the utility of ESDMs makes them a more useful method when tuning cannot be done due to limited data or a lack of understanding of the mechanistic effect variables might have on species distributions (Hao et al., 2020). This issue is of particular note with the current study, where both occurrence data and information on each taxon were limited. Marini et al. (2010) found that ESDMs could be used to predict the potential distribution of seven tropical bird species with as few as 10 observations. Our results mirror those by Marini et al. (2010), as we were able to generate single model SDMs and ensemble models with excellent fit, that is, AUC (>0.9) and TSS (>0.8), and acceptable accuracy for species with at least 10 occurrence records, but not those species with less than five observations (BRT needed at minimum 20 observations to consistently run). Additionally, our results showed the similar model performance to studies with far more observations and predictive layers (Hopkins, 2009; Wilson et al., 2011).

Conditions in the landscape are known to affect instream habitat, which in turn, can shape species occupancy (Daniel & Brown, 2013; Newton et al., 2008; Poff, 1997). In our study, precipitation during the warmest quarter or mean temperature during the driest quarter was the most important predictor of habitat suitability for 6 of the 11 species assessed. Specifically, we found areas with higher precipitation during summer months harbored greater mussel diversity compared to areas where summer precipitation was reduced. This relationship likely illustrates the effect of low flows on mussels during thermally stressing events such as periods of low precipitation. It is well known that elevated water temperatures can affect mussel survival (Khan et al., 2019, 2020; Pandolfo et al., 2010), and these effects often occur during periods of reduced flow (Archambault et al., 2014). Reduced flows are inherent to a rivers flow regime and are biologically important (Biggs et al., 2005; Bovee, 1986; Poff et al., 2006), but they can become problematic during periods of reduced precipitation and/or overuse by humans (Golladay et al., 2004; Randklev et al., 2018). Golladay et al. (2004) found significant declines in mussel fauna where a stream reaches ceased to flow, which was exacerbated by increased irrigation draws during drought. For the PRB, it remains unknown if low precipitation, water consumption, or a combination of both are contributing to mussel distribution or absence. However, given the role both have played in mussel declines elsewhere in North America, our finding should serve as a warning to conservationists and resource managers in this basin.

We also found that elevation was an important determinant of occupancy for all 11 species in this study. The elevation is known to shape mussel occurrence by affecting stream flows, water level, scouring events, and access for larval mussels to host fish (Hastie et al., 2001; Wilson et al., 2011). The latter is of note because freshwater mussels possess a unique life cycle involving an obligate parasitic larval stage (glochidia or lasidia in the case of the Mycetopodidae) that must attach to aquatic vertebrates (primarily fish) to develop into a free‐living juvenile (Barnhart et al., 2008), which also means their dispersal is tied to that of their host fish. The elevation is rooted in past geological events, which are known to influence patterns of aquatic biodiversity (e.g., Hoagstrom et al., 2014). In our study, known observations, predicted presence, and diversity are situated between the coastal fall line, which represents the maximum extent of the Gulf of México during the Cenozoic period, and the arid central plateau. The Gulf of México has slowly retreated since the Cenozoic period (Galloway et al., 2011; Smith & Bermingham, 2005), which has likely isolated taxa once connected by fish infected with glochidia or lasidia that migrated along coastal inlets or between pirated stream systems. The arid central plateau exhibits a marked decrease in precipitation compared to the lower coastal reaches of the PRB (Hudson, 2003), has a limited number of spring‐fed stream systems, and is characterized by highly variable temperatures with summer temperatures reaching upwards of 30°C. These factors have likely constrained mussel occurrence and expansion by limiting fish infected with glochidia through physical barriers such as sharp increases in slope and shifts in geology and constraining habitat available for colonization due to reductions in water quantity, quality, and colonization of stream reaches that extend to the plateau.

We found land‐use contributed very little to model performance, which is interesting given that land‐use change is often cited as a primary factor responsible for mussel declines (Allan, 2004; Box & Mossa, 1999; Randklev et al., 2015). We suspect this finding is due to the coarse resolution of our land use data and homogeneity of land use across the landscape. We also suspect it could be due to differences in scale of land‐use changes between regions where mussels have been well‐studied (e.g., United States and Europe) and the PRB. In the United States, the landscape has been altered at a scale (i.e., time and space) that is much broader and more intensive than what we observed in the PRB. In the United States, these alterations have left many streams and rivers devoid of any sort of significant mussel fauna (Haag & Williams, 2014). In contrast, the fauna in the PRB, at least in the zone above the Fall Line, which is largely comprised of small‐scale farming villages, is largely intact with almost all fauna known historically from the basin being found during recent expeditions (2017 and 2018). In coastal areas within the PRB land use is more altered (i.e., sugarcane and citrus; Hudson et al., 2005), but streams and rivers in these areas have not been well‐sampled. Therefore, it is uncertain whether or how the fauna has been affected by these activities. These differences in land use impacts between the PRB and those of river systems in the United States, Europe, or other well‐studied regions provide opportunities to better understand how aquatic communities are shaped and how populations function without the backdrop of intensive, widespread human impacts.

### 4.2 Conservation implications

We successfully predicted the occupancy of mussels in a region of México where the fauna is largely unknown. Future studies could build off these efforts through ground‐truthing our predictions utilizing methods laid out in Randklev et al. (2015) for model validation. In that study, the authors classified unsampled reaches using probabilities of occupancy into the following classes: 0%–20%, 20%–40%, 40%–60%, 60%–80%, and 80%–100%. The authors then randomly generated sample points within each probability class and performed surveys at those locations. The new survey data collected by Randklev et al. (2015) was then fed back into their models to improve model performance. Given the influence of temperature and rainfall in our study, both of which will be affected by changing climates, the models and baseline data presented in this study could be used to predict the effects of future climate conditions on freshwater biodiversity in general, and specifically, freshwater mussels. These efforts could be enhanced by including sites and reaching specific variables. For example, Wilson et al. (2011) used SDMs to determine conservation and restoration areas for *Margaritifera margaritifera* (Freshwater Pearl Mussel) found that soil clay content and carbon content were important for predicting its occurrence. Similarly, Hopkins (2009) in a study on the effects of landscape metrics and multiscale data on *Theliderma cylindrica* (Rabbitsfoot mussel) in the Ohio River system found that soil composition and geomorphic factors were highly correlated with occurrences. These examples, along with our study, could guide future modeling efforts in the PRB, and other basins, to better understand how habitat at various scales shapes mussel occupancy.

Given that species, occupancy is an interplay between species traits and selective characteristics of habitat (Poff, 1997), the inclusion of species trait information in future modeling efforts will help with understanding mechanisms behind species occurrence and optimize model prediction. Unfortunately, very little if anything is known about the life history of mussels in the PRB, which is a problem for mussel conservation and management regardless of geographic location. Because of the lack of data, future studies could focus on enumerating demographic traits such as fecundity, longevity, host use, and growth rates known to be useful for explaining occupancy for other aquatic taxa (Mims & Olden, 2012; Winemiller et al., 2015). That said, our study develops a framework for future research studies in biodiversity research and conservation biology planning in this region and provides baseline information for a poorly understood mussel fauna considered of high conservation concern (e.g., Myers et al., 2000).

## AUTHOR CONTRIBUTIONS

**Alexander H. Kiser:** Conceptualization (Equal), Data curation (Equal); Formal analysis (Equal), Investigation (Equal), Methodology (Equal), Validation (Equal) Writing—original draft (Equal). **Kevin Cummings** Conceptualization (Equal), Data curation (Equal), Funding acquisition (Equal), Investigation (Equal), Validation (Equal), Writing—review & editing (Equal). **Jeremy S**. **Tiemann:** Data curation (Equal), Investigation (Equal), Validation (Equal), Writing—review & editing (Equal). **Chase H. Smith:** Conceptualization (Equal), Data curation (Equal), Investigation (Equal), Validation (Equal), Writing—review & editing (Equal). **Nathan Johnson:** Conceptualization (Equal), Data curation (Equal), Funding acquisition (Equal), Investigation (Equal), Validation (Equal), Writing—review & editing (Equal). **Roel Lopez:** Data curation (Equal), Investigation (Equal), Methodology (Equal), Project administration (Equal), Resources (Equal), Supervision (Equal), Validation (Equal), Writing—review & editing (Equal). **Charles R**. **Randklev:** Conceptualization (Equal), Data curation (Equal), Funding acquisition (Equal), Investigation (Equal), Methodology (Equal), Project administration (Equal), Resources (Equal), Supervision (Equal), Validation (Equal), Writing—review & editing (Equal).

## CONFLICT OF INTEREST

The authors declare that they have no conflict of interest.

## Data Availability

Mussel occurrence records are available: https://doi.org/10.5061/dryad.18931zczf and through http://mussel‐project.uwsp.edu/. Climate data available through WorldClim: https://www.worldclim.org/. Land cover data available through DIVA.GIS: https://www.diva‐gis.org/gdata.
